# Osteosynthesis with anatomic reduction after malleolar fractures improves ten years outcome: a single center trial

**DOI:** 10.1007/s00590-024-04153-9

**Published:** 2024-11-26

**Authors:** Amal Chidda, Sérgio Soares, Pedro Nogueira, Joseph M. Schwab, Moritz Tannast, Angela Seidel

**Affiliations:** 1 Department of Orthopaedic Surgery and Traumatology, Fribourg Cantonal Hospital, University of Fribourg, Chemin Des Pensionnats 2-6, 1708 Fribourg, Switzerland; 2 Department of Orthopaedic Surgery and Traumatology, Valais Hospital, Martiny, Switzerland; 3 Department of Orthopaedic Surgery and Traumatology, Inselspital, University of Bern, Bern, Switzerland

**Keywords:** Malleolar fractures, Long-term outcome, Patient reported outcome, Radiological outcome

## Abstract

**Introduction:**

Malleolar fractures are the most common ankle fractures and a major risk factor for ankle osteoarthritis in the long-term. Little is known about modifiable risk factors for a satisfactory outcome. This study aimed to assess the long-term clinical, functional and radiological outcomes in patients after osteosynthesis.

**Methods:**

In this retrospective single center study, we assessed the difference in patients who underwent surgical intervention for malleolar fractures sustained between 2007 and 2014. The reduction was assessed on the first postoperative radiograph. At follow-up patients completed a questionnaire, including the European Foot and Ankle Society (EFAS) and Short Form-12 (SF-12) scores to evaluate patient-reported outcomes and quality of life. Ankle osteoarthritis was assessed using the Kellgren and Lawrence classification.

**Results:**

One hundred seventeen patients, 102 with anatomic reduction and 15 with malreduction, were reached at mean follow-up at 11.4 years and 10.9 years. The mean EFAS score was 18,0 for anatomic and 16,1 for nonanatomic reduction and 6.1 and 4.5 for the sport component. The rate of satisfaction with the result was 8.2 in anatomic reduction and 7.5 in the malreduction. There was no significant difference in the SF-12 group between the two groups. Anatomic reduction is a protective facture for a satisfactory outcome in the univariate model with the hazard ratio of 5.94.

**Conclusion:**

Anatomic reduction is one of the strongest protective factors for satisfactory outcome after malleolar fractures in a follow-up of more than 10 years.

## Introduction

Foot and ankle fractures represent a significant percentage of orthopedic injuries, accounting for approximately 15.6% of reported fractures [1]. Among these, malleolar fractures stand as the most prevalent, comprising 10.7% of cases [1]. Despite advancements in fracture management, long-term complications persist, with up to 23% of individuals experiencing chronic pain and compromised quality of life post-treatment [11]. Moreover, individuals engaged in manual labor face notable challenges in returning to their previous occupational capacities following such injuries [16]. Risk factors like obesity, age and malreduction have emerged as determinants of diminished functional outcomes [2, 8, 13]. Yet, those risk factors are not consistent throughout different studies. A comprehensive understanding of the divergent impacts of malleolar fractures on long-term quality of life and the underlying risk factors have not been assessed.

The assessment of quality of life (QoL) following foot and ankle fractures has relied significantly on standardized measures such as the SF-12, known for its validated use across multiple European countries and its age and sex-dependent normative values [3, 18]. For more specific information on patient-reported foot function (PROMS) an additional score is needed. Since the AOFAS score is not validated, additional scores have been developed [9]. The questionnaire established by the European Foot and Ankle Society is a valuable instrument, accommodating both traumatic and nontraumatic cases and featuring sport-specific inquiries critical for evaluating physical activities and sports involvement [12].

In the long-term hindfoot fractures are a well-known risk factor for developing osteoarthritis [5, 8]. After 17 years follow-up 38% of patient with an ankle fracture have developed advanced ankle osteoarthritis on radiographs [5, 8]. Risk factors encompass multifaceted fractures, elevated body mass index, advanced age, and prolonged post-surgery duration, all of which contribute significantly to heightened OA risk [8]. Yet, those factors vary between the different studies. Understanding the degree of OA and its correlation with patient-reported outcomes becomes imperative in comprehending the impact of these fractures.

The aim of the project is to determine the long-term outcome of malleolar fractures: 1. Patient-reported outcome, 2. Quality of life and 3. Osteoarthritis. Additional risk factors for unsatisfactory outcome were assessed.

## Methods

Between 2007 and 2014 a consecutive series of 271 patients received treatment at our hospital for malleolar fractures. Patient specific data as age at time of injury, sex, smoking, diabetes and BMI were recorded. The energy of trauma, an open fractures, the delay of fixation, a temporary external fixation was noted from the chart. The quality of fractures fixation was assessed in the postoperative radiographs. Malreduction was defined as an intraarticular step > 1 mm, a shortening or lengthening of the peroneus of > 2 mm, the reduction of syndesmosis according to Pettrone [10], a widening of the medial clear space of more than 1 mm compared to the superior clear space and talar subluxation seen in the lateral radiograph as a asymmetric joint space. Reoperations and the complication rate were analyzed according to the Sink classification [14].

During the follow-up assessments, Patient-Reported Outcome Measures (PROMs) were evaluated using the EFAS score [12]. It is 10-item validated PROM score of foot and ankle injuries, including 6 questions related to pain and daily activities, and 4 questions related to sport activities. Quality of life assessments were conducted using the SF-12 [3], and the degree of osteoarthritis was determined based on follow-up radiographs employing the Kellgren-Lawrence classification [4]. The patients were asked if they were satisfied with their outcome: a Likert Scale from 0 to 10 was used.

The study is approved by the “ Ethics committee CER-VD”. The study ID is 2021-01120.

For statistics R/RStudio (The R Foundation for Statistical Computing) was used. Continuous data (EFAS score and SF12) was tested for normal distribution with the Kolmogorov–Smirnov test. As the EFAS score and SF12 were not normally distributed the Mann–Whitney U test was used. A univariate and multivariate analysis was performed to determine the risk factors and hazard ratios.

## Results

### Demographics

**Fig. 1:**
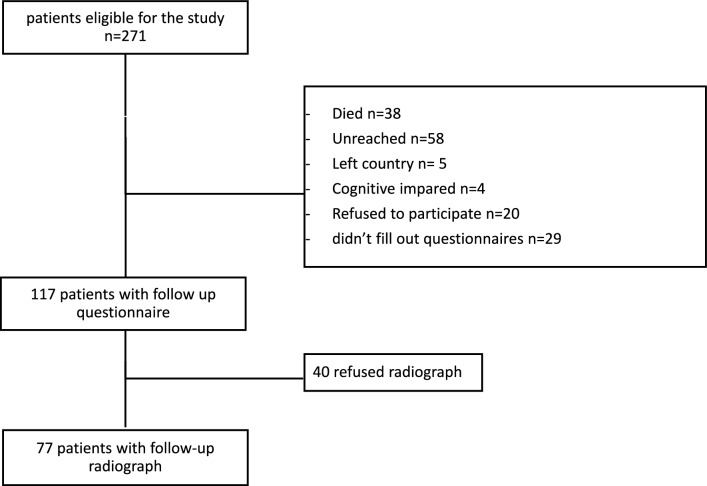
Flowchart with included patient and those lost to follow-up

One hundred seventeen patients, 102 (87%) with an anatomic reduction and 15 (13%) with a malreduction, were reached at mean follow-up at 11.3 years (Fig. 1). The average age at time of injury was 50 with a male to female ratio of 71/46. There were 30 (26%) smokers and 13 (11%) patients with diabetes. The body mass index was in average 29.2 kg/m^2 (Table 1).

**Table 1:** Demographics of the patients reached for the follow-up questionnaire at 10 years after initial surgery. The patient population was divided into groups with anatomic reduction and malreduction

		Anatomic Reduction	Malreduction	Total
N		102	15	117
Demographics				
Gender	**Male**	62	9	71
	**Female**	40	6	46
Age (in years)		50	48	
BMI		29,4	27,8	
ASA	**I**	26	4	30
	**II**	62	8	70
	**III**	5	1	6
Smoking		27	3	30
Diabetes		11	2	13
Energy at Trauma	**High**	39	6	45
Fracture description				
Fracture dislocation		47	5	52
Open fracture		2	2	4
Weber Classification	**A**	2	0	2
	**B**	77	11	88
	**C**	16	3	19
Lauge-Hansen classification	**SER III**	9	0	9
	**SER IV**	73	11	84
	**PA III**	1	0	1
	**PA IV**	6	0	6
	**PER III**	2	0	2
	**PER IV**	6	3	9
Chaput Fragment		10	2	12

Forty-five patients (38%) had high-energy trauma which led in 4 cases (3%) to an open fracture. There were 52 fractures dislocation (44%). Initial stabilization was performed with an external fixator in 4 patients. No difference of the groups was found in either the Weber or the Lauge-Hansen classification. Additionally, the baseline data did not show any significant differences except the fact that the group which had a malreduction had 10 times more open fractures and fractures initially treated with an external fixator.

In the malreduction group 15 patients an intraarticular step of > 1 mm was found in 5 patients. The postoperative radiographs showed a fibula shortening in 3 patients and a syndesmotic malreduction in 7 patients. There was one patient with a widening of the medial clear space.

## PROMS, EFAS score

The mean EFAS score was 18.0 in the group with anatomical reduction and 16.1 in the group with malreduction. For the sport component the score was 6.1 and 4.5, respectively. The average rate of satisfaction was 8.2 in the anatomical reduced group and 7.5 in the group with a malreduction (Table 2).

**Table 2:** Outcome of the patient reached for the 10 year follow-up divided into the group patients with anatomic reduction and malreduction

		Anatomic Reduction	Malreduction
N		102	15
Outcome			
Mean follow-up time (years)		11,4	10,9
PCS		48,0	49,0
MCS		55,5	54,6
EFAS		18,0	16,1
EFAS-Sport		6,1	4,5
Actual OA	**I**	20	0
	**II**	38	7
	**III**	5	1
	**IV**	3	3
Satisfaction		8,2	7,5
Arthrodesis		1	1
Total ankle replacement		1	1

## Quality of life

The mean SF 12 physical component was 48.0 for the anatomical reduced group and 49.0 for the nonanatomic reduction group. This is in average slightly lower than the mean score of, for example, the German population (50.7) [3]. This is different to the SF 12 mental component with the average score of 55.5 in the anatomical reduced group and 54.6 in the malreduction group. This is higher than the mean German score (52.3) [3] (Table 2).

## Osteoarthritis

Seventy-seven patients (66%) underwent a follow-up radiography and the distribution of Kellgren-Lawrence levels of osteoarthritis was: 3 patients (5%) with Level IV in the anatomical reduced group, and 3 (27%) in the group with malreduction, 5 patients (8%) and 1 patients (9%) with Level III, 38 patients (58%) and 7 patients (64%) with Level II, 20 patients (30%) and 0 patients (0%) with Level I. There was one arthrodesis and one total ankle replacement in each group (Table 2).

## Reoperations

The most common reoperation is hardware removal which was performed in 60% of cases. The endpoint of a fusion was reached in 2 patients and an ankle replacement was done in 2 other patients.

## Risk factors for unsatisfactory outcome/failure

**Fig. 2:**
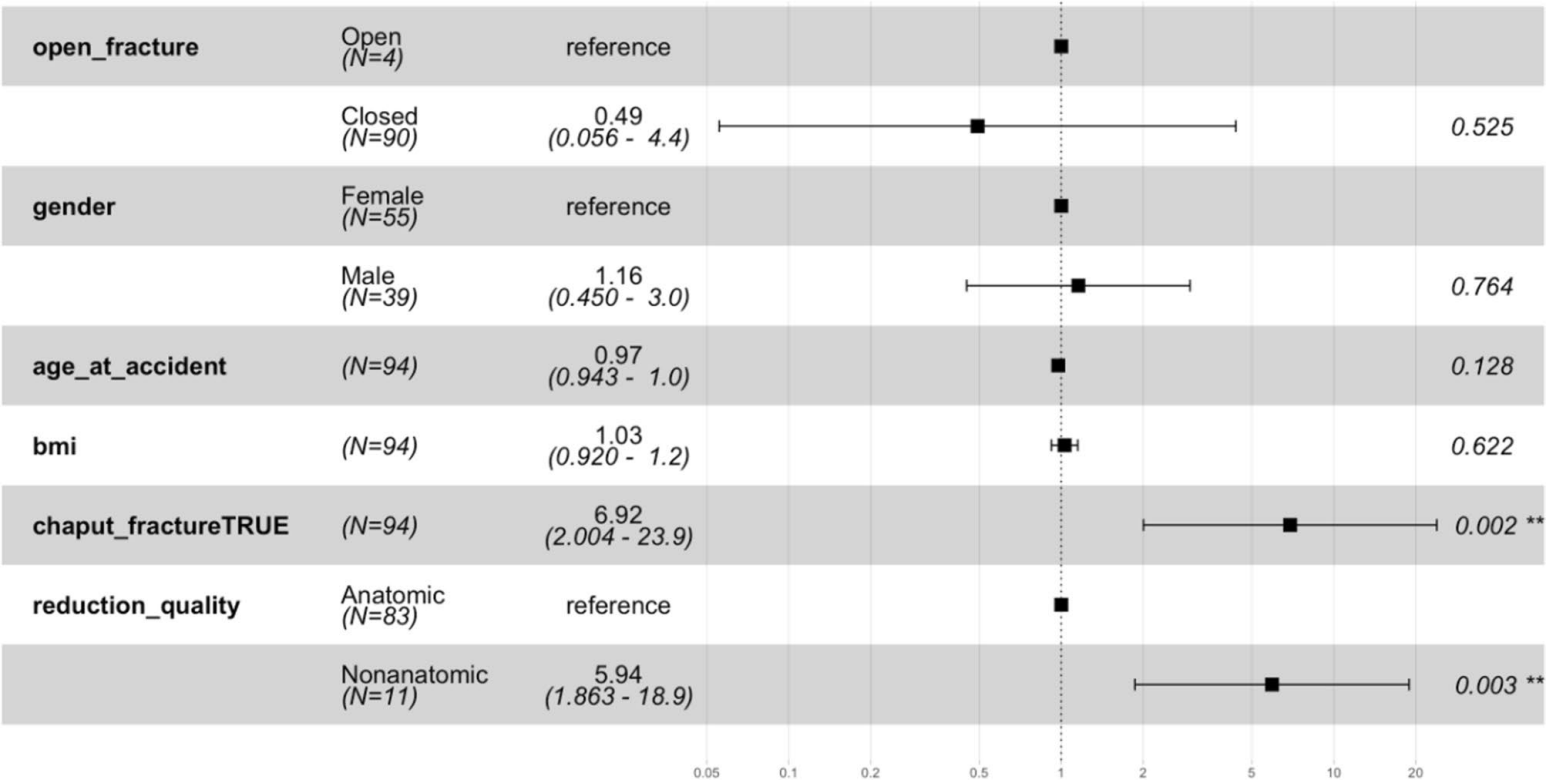
Hazard ratio and confident interval for different factors for unsatisfactory outcome. The significant risk factors are malreduction and the fixed Chaput fragment

Unsatisfactory outcomes were defined as total ankle replacement, ankle fusion, ankle osteoarthritis Level III or IV or a satisfaction score of < 6 in the Likert scale according to the patient. Anatomic reduction is a protective factor in the univariate model with a hazard ratio of 5.94 (p-value = 0.003). Another risk factor found was a Chaput fragment needing fixation with a hazard ratio of 6.92 (p = 0.002) (Fig. 1). Additionally, age at the time of injury seems to be relevant, as younger patients had a worse outcome (Fig. 2).

## Discussion

The outcome 10 years after ankle fractures can be assessed in different ways. Unsatisfactory outcomes should be considered as reduced patient-reported outcome, reduced quality of life, advanced ankle osteoarthritis in the radiograph or ankle fusion and ankle replacement. All these factors should be analyzed if risk factors are assessed. In our study population the strongest and only risk factor that can be affected was the malreduction after the surgery.

In the literature different risk factors are found for unsatisfactory outcome. Most of those risk factors were only seen in one study population but could not be confirmed in another study population. This might be considered as unspecific confounder.

## Patient-reported outcomes

Roberts at all identified the main factors for worse patient-reported outcome the malreduction [13]. Malreduction was assessed on postoperative radiographs and for evaluating the patient-reported outcome the Olerud-Molander Ankle Score (OMAS) was used. They divided their study population in 88 patients with malreduction and 107 patients without malreduction [13]. At minimum follow-up of 6 years, the OMAS was on average 57.3 in the malreduction group and 71.2 in the reduced group, with higher scores indicating better outcome. This is in line with our study population in which an unsatisfactory outcome was more likely found in the group with the malreduction. A more complex fractures with more malleoli involved had a higher malreduction rate and a worse OMAS score in the study by Roberts et al. [13]. This contrasts with the findings of Verhage et al. which could not find any difference between bimalleolar and trimalleolar fractures in a case series of 243 patients and a follow-up of 9.6 years [17]. To evaluate the patient-reported outcome they used the American Academy of Orthopedic Surgeon (AAOS) score. Another risk factor for worse outcomes in literature was a BMI. In their case series of 478 patients at a follow-up of 11.1 years the Manchester Oxford foot and ankle questionnaire showed an important different in the obese patient population (BMI > 30 kg/m^2^) compared to the normal weight patient (BMI < 25 kg/m^2^), for pain (mean 18.7 vs 33) and function (mean 12.5 vs 27.3), having the higher score indicating better results [7]. The BMI as a risk factor could only be found in the study by Cardosa et al. and was not reproduced in our study [2].

## Quality of life

The study of Cardoso et al. assessed the quality of life with the SF-12 11 years after ankle fractures [2]. They found that an average SF-12 physical component in the normal weight/obesity group 48.7/43.9 and the SF.12 mental component 47.2/44.4. In their conclusion a higher BMI at time of injury is a predictor for worse long-term outcome which has an important effect on their quality of life [2]. This could not be confirmed by our study population. The SF-12 score did not show any difference in either study groups. Additionally, the mental component of the score was with 55 much higher than Cardoso´s study population and the normal value of most countries. Our results suggest that the quality-of-life scores might not be specific enough to assess the outcome after ankle fractures and a foot and ankle specific patient-reported outcome is needed.

## Ankle osteoarthritis rate

In the long-term hindfoot fractures are a well-known risk factor for developing osteoarthritis. The study by Luebekke et al. assessed the rate of advanced stage osteoarthritis, classified by the Kellgren and Lawrence classification stage 3 and 4. The included 102 malleolar fractures with an average follow-up of 18 years showed a radiographic OA rate of 38% [8]. Risk factors are complex fractures, increasing body mass index, age, and length of time since surgery [8]. In another study, 109 patients were seen at average follow-up of 12.9 years, which had a routine arthroscopic evaluation during the initial fixation of an ankle fractures. The Kannus arthritis score was used to evaluate the OA rate on follow-up radiographs. This is a 100-point scale evaluating joint space narrowing, osteophytes, calcification of ligaments and cyst formation [6]. A score of < 90 is defined as poor outcome and was used to describe OA6. Cartilage lesions found in arthroscopy during fixation of the ankle fractures are associated with symptomatic OA with an odds ratio of 3.5 to develop symptomatic OA [15]. This might be in line with the study of Luebekke et al. as complex fractures might be associated with a higher rate of cartilage lesions as well as a higher rate of OA [8, 15]. In our study population the rate of advanced OA in the malreduction group was 27% and much higher than in the group with anatomic reduction (9%). The assumption that in the malreduction group were more complex fractures could not be confirmed.

In different studies the rate of failure due to ankle OA is between 1.2% and 2% [2, 17]. The rate to ankle fusion or ankle replacement was 1.2% in the study of 243 patient with an average follow-up of 9.6 years after malleolar fractures osteosynthesis [17]. This rate was slightly higher in the study by Cardoso et al. of 255 patients which had a rate of 1% for ankle fusion and 1% of ankle replacement at 11-years after malleolar fractures osteosynthesis [2]. This mirrors our study with a failure rate of 3% at a 10 year follow-up. Even if we included different types of fractures the expedited rate 11 years after the injury is 1–3% in all studies. This information can be provided to the patients. The percentage of symptomatic OA is estimated to increase over time. Time to failure is on average 21 years after a malleolar fracture [5]. Postoperative complication, severity of fractures and age at the time of injury shorten this time [5].

## Reoperation rate

Our reoperation rate of 63% is higher than in the case comparison study of Karkkola et al. which noticed a reoperation rate of 30% [7]. This is due to our high hardware removal rate of 60%. This rate is the double of most studies who remove the hardware in 27 to 29% [7, 17]. Even though the hardware removal is not performed routinely, most patients feel bothered by the fibular plate and request surgery. The indication for hardware removal should be reconsidered.

## Limitations

A well-known limitation of the retrospective study design is the high rate of 57% of loss of follow-up. This can be a common problem for studies with long-term follow-up. We have included the relevant data on our entire cohort for analysis, but we realize this data is not complete. Another limitation in the assessment of malreduction on the first postoperative radiograph. A scanner or for the syndesmotic malreduction a contralateral radiograph would be more accurate. But those examens were not performed at our clinic 10 years ago. In addition, the two study groups are inequal of size. This might influence the statistical analysis. In contrast it represents the malreduction rate of 13%. There are inherent limitations of a retrospective review over a long-term period such as multiple surgeons being involved. Yet, nobody of the study group was initial involved in the surgery.

## Conclusion

In the long-term the rate is 3% for ankle arthrodesis; ankle replacement and 16% for advanced ankle osteoarthritis after malleolar fractures. Anatomic reduction is one of the strongest protective factors for satisfactory outcome after malleolar fractures in a follow-up of more than 10 years.
